# Comment on “Triplet chemotherapy combined with anti-EGFR treatment in RAS wild-type colorectal cancer: a network meta-analysis”

**DOI:** 10.1093/oncolo/oyag290

**Published:** 2026-07-25

**Authors:** Sara Cherri, Alberto Zaniboni

**Affiliations:** Department of Oncology, Fondazione Poliambulanza, Brescia, Italy; Department of Oncology, Fondazione Poliambulanza, Brescia, Italy

Dear editor,

We read with interest the network meta-analysis by Di Nardo et al. evaluating the role of triplet chemotherapy combined with anti-EGFR agents in RAS wild-type metastatic colorectal cancer.^1^

While the authors report no significant improvement in response-related endpoints with treatment intensification, the pooled OS analysis favored triplet chemotherapy plus anti-EGFR treatment (HR 0.82, 95% CI, 0.70-0.97; *P* = .022). However, the magnitude of the OS benefit appears relatively modest, with hazard ratios close to unity and a limited absolute gain across studies.

In this context, recently reported long-term results from the phase III TRIPLETE study provide relevant additional evidence.^2^ With a median follow-up exceeding 5 years, upfront mFOLFOXIRI plus panitumumab demonstrated a statistically significant OS advantage compared with mFOLFOX plus panitumumab, with a median OS of 41.1 months versus 33.3 months (HR 0.79, 95% CI, 0.63-0.99).

Importantly, this survival benefit was observed despite no differences in objective response rate (75% vs 78%), progression-free survival (12.7 vs 12.4 months), early tumor shrinkage, or depth of response. We note that the long-term survival outcomes should be interpreted with caution, given their discordance with the early efficacy endpoints, as it is not immediately clear how the absence of improved initial disease control could translate into a delayed survival benefit.

Moreover, the progressive separation of OS curves over time and the longer post-progression survival (24.6 vs 17.7 months) may suggest a delayed effect; however, alternative explanations such as post-progression treatments or potential selection biases cannot be excluded.

Taken together, these observations underscore the importance of mature follow-up while maintaining caution in the interpretation of survival signals in the absence of benefit in the primary endpoint (objective response rate).

Sincerely,

Sara Cherri, MD

Alberto Zaniboni, MD
